# Predictive value of inflammatory markers for cancer diagnosis in primary care: a prospective cohort study using electronic health records

**DOI:** 10.1038/s41416-019-0458-x

**Published:** 2019-04-24

**Authors:** Jessica Watson, Chris Salisbury, Jonathan Banks, Penny Whiting, Willie Hamilton

**Affiliations:** 1 Population Health Sciences, Bristol Medical School, University of Bristol, Bristol, UK; 2 NIHR CLAHRC West, Bristol, UK; 3 University of Exeter Medical School, Bristol, UK

**Keywords:** Diagnostic markers, Cancer, Diagnosis

## Abstract

**Background:**

Early identification of cancer in primary care is important and challenging. This study examined the diagnostic utility of inflammatory markers (C-reactive protein, erythrocyte sedimentation rate and plasma viscosity) for cancer diagnosis in primary care.

**Methods:**

Cohort study of 160,000 patients with inflammatory marker testing in 2014, plus 40,000 untested matched controls, using Clinical Practice Research Datalink (CPRD), with Cancer Registry linkage. Primary outcome was one-year cancer incidence.

**Results:**

Primary care patients with a raised inflammatory marker have a one-year cancer incidence of 3.53% (95% CI 3.37–3.70), compared to 1.50% (1.43–1.58) in those with normal inflammatory markers, and 0.97% (0.87–1.07) in untested controls. Cancer risk is greater with higher inflammatory marker levels, with older age and in men; risk rises further when a repeat test is abnormal but falls if it normalises. Men over 50 and women over 60 with raised inflammatory markers have a cancer risk which exceeds the 3% NICE threshold for urgent investigation. Sensitivities for cancer were 46.1% for CRP, 43.6% ESR and 49.7% for PV.

**Conclusion:**

Cancer should be considered in patients with raised inflammatory markers. However, inflammatory markers have a poor sensitivity for cancer and are therefore not useful as ‘rule-out’ test.

## Background

Cancer is common—affecting 50% of the UK population during their lifetime.^1^ Early diagnosis is important, with delayed diagnosis associated with more advanced stage at diagnosis and decreased survival.^2,3^ Cancer diagnosis in primary care can be challenging; many of the early symptoms are non-specific and can be difficult to differentiate from the symptoms of common benign conditions. When patients present with high-risk symptoms, GPs in England can refer via urgent cancer referral pathways, but many patients with cancer present with low-risk (but not no-risk) symptoms.^4–6^ GPs need to triage patients with low-risk symptoms to identify those needing further investigations, using additional ‘clues’ from history, examination and investigations. One triaging tool increasingly used in clinical practice is inflammatory marker tests, with the three most commonly used in UK practice being C-reactive protein (CRP), erythrocyte sedimentation rate (ESR) and plasma viscosity (PV). These are often performed as a ‘rule-out’ test by clinicians trying to exclude serious underlying disease, including cancer.^7^ This practice is largely unsupported by evidence; inflammatory markers are not recognised within current guidelines for cancer diagnosis,^8^ with the exception of myeloma, where first line tests include ESR or PV. Cohort studies in the general population (irrespective of symptoms) have examined the association between raised CRP and risk of future cancer,^9–11^ including meta-analyses of the risk of future colorectal,^12,13^ lung,^14^ ovarian^15^ and breast cancer.^16,17^ However, the associations are not strong enough to be clinically useful for identification of symptomatic cancer. Studies of specific cancers in symptomatic primary care patients have reported an association between raised inflammatory markers for bladder and kidney cancers, Hodgkin and non-Hodgkin lymphomas, plus myeloma, albeit with very low positive predictive values (PPVs) for a raised inflammatory marker result.^18–23^

To our knowledge, there have been no studies to measure the overall clinical utility of inflammatory markers for cancer diagnosis in primary care. This study aimed to address this omission by determining the predictive value of inflammatory markers for cancer diagnosis in primary care; measuring cancer incidence in those with normal and raised inflammatory markers and calculating measures of diagnostic accuracy.

## Methods

### Data sources

This was a prospective cohort study of UK primary care patients using routinely collected data from electronic health records in the Clinical Practice Research Datalink (CPRD). This contains anonymised, coded data on primary care consultations, laboratory results and referrals.

### Participants

Participants were 160,000 patients, aged ≥18, of either sex, who had a primary care inflammatory marker blood test (CRP, ESR or PV) taken in 2014, selected at random by CPRD.

A comparison sample of 40,000 patients with no inflammatory markers taken during 2014 was matched by age (within 5-year bands), sex and practice to a random subset of 40,000 patients from the inflammatory marker test group. This group, whilst not needed for the diagnostic test accuracy evaluation, allows us to compare the incidence of disease in the tested vs. untested populations. Given that inflammatory marker tests are more likely to be done in symptomatic patients at higher risk of cancer than the general population, this gives us an indication of the diagnostic accuracy of clinicians’ gestalt judgement to perform an inflammatory marker test.

Linked data from the English Cancer Registry was obtained by the CPRD for 110,245 patients. Patients in whom it was not possible to obtain linked data were resident outside England, lacked a valid NHS identifier, were registered at a GP practice which had not consented to linkage or were individuals who had personally dissented from linkage.

Patients with a record of cancer in CPRD or the cancer registry in the 2-year period before the index date were excluded (*n* = 4489), as were patients who had missing or spurious inflammatory marker test results (*n* = 662).

### Index tests

Three inflammatory marker tests were analysed: CRP, ESR and PV. The index date was defined as the first date of inflammatory marker blood testing in 2014; controls were allocated the same index date as their matched case. As different laboratories use different thresholds to define abnormality, we used the mean upper limit of normal from laboratories within our study to define a raised inflammatory marker. For CRP this mean was 6.8 mg/l, therefore we rounded to a conservative threshold of 7 mg/l. For PV the upper limit of normal was 1.72 mPa.s. For ESR, we used the mean reported upper limit of normal, stratified by gender and in 10-year age bands, which varied from 11 mm/h for women under 40, to 23 mm/h for men over 80 (see supplementary table 1). For those with the same inflammatory marker test coded more than once on the same day (*n* = 231), the highest value was retained. For patients with more than one type of inflammatory marker on the same day a binary variable was generated for ‘any raised inflammatory marker’, which was positive if any of CRP, PV or ESR were raised.

### Outcome variables

The primary outcome was the one-year cancer incidence; we also examined two-year incidence, lest there be a delayed effect. All new cancer diagnoses, excluding benign and in situ cancers and non-melanomatous skin cancers, were identified by searching the CPRD records for any of the 2120 cancer-related codes (available from the authors on request). This code list has been developed using published methods^24^ and validated in several studies: it is collated into 22 common cancer sites. New cancer diagnoses were also extracted from the cancer registry by CPRD staff. Patients were included as having cancer if a diagnosis was recorded in either CPRD or the cancer registry, with the earliest record of cancer assigned to be the date of diagnosis.

In order to identify the main symptoms associated with inflammatory marker testing, we searched the CPRD for codes in the 28 days prior to and including the index date. We retained the 200 most frequently occurring codes in those with raised inflammatory markers, normal inflammatory markers and untested controls. After removing non-clinical administrative codes we used frequency counts to identify the most commonly occurring symptoms, categorised according to the International Classification of Primary Care.^25^ We then used methods described previously^24^ to generate complete code lists for each of these symptoms.

### Sample size calculation

Assuming a 2:1 ratio of normal to raised inflammatory markers, to detect an increase of 0.2% cancer incidence in the test positive compared to test negative group, assuming a baseline (test negative) condition incidence of 0.8% requires a sample size of 77,577 for 80% power and alpha = 0.05. The CPRD were willing to offer a sample size of 160,000 which provided a power of 98% for the same estimated differences and allowed subgroup analyses. The focus of the study was cancer outcomes in those with inflammatory marker testing, therefore the untested group—used as a benchmark for the tested group—was deliberately kept small to ensure maximum power in the main study.

### Analysis

The primary analysis reported the one-year cancer incidence (hereafter referred to as ‘cancer incidence’) for patients with raised vs. normal inflammatory markers, and vs. untested patients. For those with raised inflammatory markers, this is equivalent to the PPV. Sub-analyses stratified cancer incidence by gender and age in 10-year bands. We also stratified analysis according to whether multiple inflammatory markers showed concordant or discordant results, whether repeat inflammatory markers were normal or abnormal, and according to symptoms recorded in CPRD in the 28 days prior to testing. We used logistic regression to examine the dose-response relationship between CRP, ESR and PV test results as continuous variables, and cancer diagnosis as a binary variable, also generating a receiver-operating characteristic (ROC curve). Logistic regression was also used to generate diagnostic odds ratios (DOR), which were adjusted for age and gender. Several sensitivity analyses in subgroups were performed; excluding those with <1 year follow-up in CPRD (for example patients moving practice), excluding those with pre-existing autoimmune disease and recent infections (who might be expected to have tests for monitoring purposes) and excluding those with myeloma (as this is already known to have a strong association with raised inflammatory markers). We also performed a sensitivity analysis using the laboratory’s own upper limit of normal rather than our own derived thresholds. A final sensitivity analysis explored the impact of restricting analysis only to those patients eligible for cancer registry linkage.

The reporting of this study conforms to the STARD^26^ and RECORD^27^ statements. Analysis was performed using Stata15, using the module DIAGT^28^ to calculate summary statistics from 2 × 2 tables including sensitivity, specificity, PPV and negative predictive value (NPV).

## Results

**Fig. 1:**
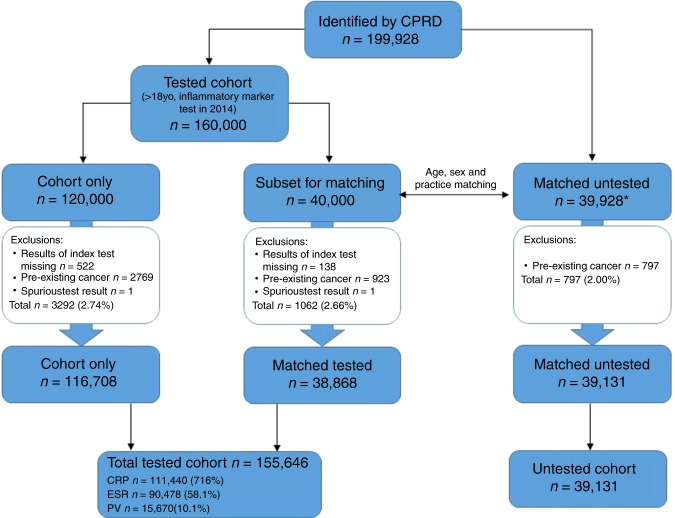
Flowchart showing participants and exclusions. *Matched untested group consists of 39,928 because from the 40,000 patients from the cohort who were randomly selected for matching, 72 had no suitable age, sex and practice matched control

After exclusions (see Fig. 1), the inflammatory marker cohort contained 155,646 patients; of these 111,440 (71.6%) had a CRP test, 90 478 (58.1%) had an ESR test, and 15,670 (10.1%) had a PV test. 61,545 (39.5%) had more than one test performed together on the index date, mostly CRP and ESR (50 522), followed by CRP and PV (10 494). 46,092 (29.6%) of the tested cohort had at least one raised inflammatory marker.

**Fig. 2:**
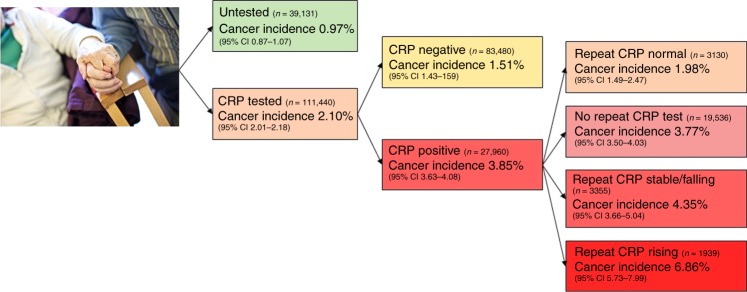
Flowchart of one-year cancer incidence according to CRP test results. The right-hand column shows cancer incidence according to repeat test result; defined as the first CRP test performed in the 3 months following the index date

Table 1 shows the gender, age and cancer incidence of the cohort, grouped by test result. The overall incidence of cancer for those with a raised inflammatory marker was 3.53% (95% CI 3.37–3.70), compared to 1.50% (1.43–1.58) with normal inflammatory markers (*p* < 0.001). The untested cohort had a cancer incidence of 0.97% (0.87–1.07%). Most cancer diagnoses were made within 1 year of testing, with no evidence of significantly increased cancer risk in the second year after a raised inflammatory marker compared to untested controls. For each of the three tests, sensitivities, specificities, PPV, AUC and DOR for a positive result are shown in Table 2. Figure 2 shows cancer incidence according to test results in a flowchart for CRP, the most frequently used of the three inflammatory marker tests. A logistic regression model containing age and gender had an AUC of 0.736, compared to 0.747 for a full model containing age, gender and CRP test result as a continuous variable (*p* < 0.001); 0.759 with age, gender and ESR (*p* < 0.001); and 0.760 with age gender and PV (*p* < 0.001).

**Table 1:** Demographics and cancer incidence by test result

	Raised inflammatory markers^a^	Normal inflammatory markers^b^	Untested
Number of patients (*n*)	46 092	109 554	39 131
Female (%)	64.9	61.1	62.0
Age, years (mean, IQR)	61.9 (46.5–75.3)	53.9 (39.9–68.5)	56.2 (41.6–70.7)
Cancers diagnosed in 1 year (*n*)	1 629	1 648	379
1-year cancer incidence % (95% CI)^c^	3.53 (3.37–3.70)	1.50 (1.43–1.58)	0.97 (0.87–1.07)
Second year cancer incidence % (95% CI)	1.07 (0.97–1.16)	0.77 (0.72–0.82)	0.96 (0.86–1.05)

^a^One or more inflammatory marker raised

^b^All inflammatory markers tested normal

^c^Equivalent to positive predictive values (PPV) for the test positive group

**Table 2:** Performance characteristics of inflammatory markers tests for cancer

	True positives (*n*)	False positives (*n*)	True negatives (*n*)	False negatives (*n*)	Sensitivity	Specificity	AUC^a^	DOR^b^ (unadjusted)	DOR (adjusted for age + gender)
CRP (*n* = 111,440)	1077	26,883	82,219	1261	46.1% (44.0–48.1)	75.4% (75.1–75.6)	0.641 (0.63–0.65)	2.29^*^ (2.12–2.46)	1.79^*^ (1.66–1.93)
ESR (*n* = 90,478)	810	21,628	66,993	1047	43.6% (41.4–45.9)	75.6% (75.3–75.9)	0.644 (0.63–0.66)	1.98^*^ (1.83–2.15)	1.75^*^ (1.61–1.90)
PV (*n* = 15,670)	153	4289	11,073	155	49.7% (44.0–55.4)	72.1% (71.4–72.8)	0.631 (0.60–0.66)	1.69^*^ (1.43–1.99)	1.45^*^ (1.22–1.71)

^*^p < 0.0001

^a^Area under receiver operator curve

^b^Diagnostic odds ratio

Sensitivity analyses are shown in supplementary table 2; these gave minor differences in results which were not clinically significant. Restricting analysis to the 110,245 patients eligible for Cancer Registry linkage increased cancer incidence marginally to 3.82% (3.58–4.05) in the raised inflammatory marker group, 1.63% (1.53–1.73) in the normal inflammatory marker group and 1.04% (0.90–1.17) in the untested group.

### Inflammatory marker levels

The incidence of cancer increased with rising inflammatory markers with a dose-response relationship; see supplementary Fig. 1. Out of 506 people with ESR ≥ 100, 69 (13.6%) developed cancer in 1 year; with CRP ≥ 100 (*n* = 1983), 135 (6.81%) developed cancer; with PV ≥ 2.0 mm/h (*n* = 342), 31 (9.06%) developed cancer.

### Repeat testing

When patients with a raised inflammatory marker (*n* = 46,092) had a second inflammatory marker test taken within 90 days (*n* = 13,873), the cancer incidence was greatest if the second test result was further increased, at 6.86% (5.73–7.99) for CRP, 5.04% (4.01–6.08) for ESR and 4.13% (2.00–6.26) for PV. If the second test was lower than the first, but still above the normal range, the cancer incidence was 4.35% (3.66–5.04) for CRP, 3.55% (2.86–4.24) for ESR and 3.28% (1.79–4.78) for PV. If the repeat test was normal the cancer incidence fell to 1.98% (1.49–2.47) for CRP, 2.49% (1.73–3.26) for ESR, and 1.32% (0.34–2.29) for PV. This is shown in Fig. 2 for the most frequently used inflammatory marker, CRP.

### Multiple inflammatory marker tests

For the 61,545 who had more than one inflammatory marker test done together, if both were normal (*n* = 39 368; 64.0%), the cancer incidence was 1.28% (1.17–1.39), with one raised and the other normal (*n* = 13 472; 21.9%) the cancer incidence was 2.27% (2.02–2.52), if both were raised (*n* = 8 705; 14.1%) then cancer incidence was 4.71% (4.26–5.16).

### Effects of age and gender

**Fig. 3:**
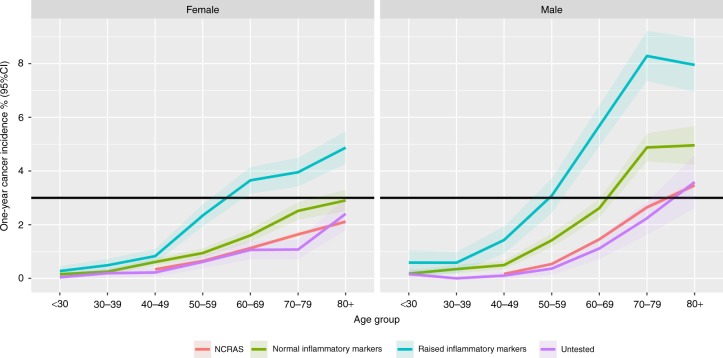
One-year incidence of cancer, stratified by age and gender. Red line, NCRAS, Cancer incidence from UK National Cancer Registration and Analysis Service; green line, normal inflammatory markers, *n* = 109,554; blue line, raised inflammatory markers, *n* = 46,092; purple line, untested, *n* = 39,131. Shaded areas = 95% confidence intervals. The black line represents the 3% threshold used by NICE for urgent investigation or referral

Breakdown by age and gender (Fig. 3) shows that cancer incidence increases with age and is higher in men, exceeding the NICE 3% threshold for urgent cancer referral for men over 50 and women over 60 years with a raised inflammatory marker, who have a cancer incidence of 6.44% (6.00–6.88) and 4.22% (3.90–4.45) respectively. For women under the age of 60 with a raised inflammatory marker, cancer incidence was 1.20% (1.03–1.37); for men under 50 it was 1.03% (0.72–1.33). Patients with normal inflammatory markers have a cancer incidence which exceeds that of untested controls and national cancer registry rates but is lower than the raised inflammatory marker group. In particular, men over 60 with *normal* inflammatory markers have a cancer incidence which exceeds the NICE 3% threshold.

### Cancer sites

The types of cancer are shown in Table 3; the cancer sites broadly reflect overall cancer incidence in 2014 from National Cancer Registry figures, apart from breast cancer and prostate cancer, which are notably less frequent in the raised inflammatory marker group. Myeloma contributed only 45 out of 2145 cancers in the raised inflammatory marker group; sensitivity analysis demonstrated minimal difference in overall results when these were excluded (cancer incidence in the raised inflammatory marker group 3.45% excluding myeloma, vs. 3.53% overall; supplementary table 2).

**Table 3:** Types of cancer diagnosed by gender in those with raised inflammatory markers, compared to types of cancer diagnosed nationally in England in 2014^35^

	Male		Female	
	Raised inflammatory markers	National incidence	Raised inflammatory markers	National incidence
*Cancer site*	% (*n*)	%	% (*n*)	%
Bladder	3.91 (41)	4.14	1.59 (17)	1.56
Breast	0.19 (2)	0.22	15.78 (169)	31.56
Cervix	–	–	1.12 (12)	1.77
Head and neck	1.34 (14)	1.56	0.19 (2)	1.66
Kidney	3.82 (40)	3.79	2.80 (30)	2.34
Leukaemia	2.10 (22)	3.25	2.43 (26)	2.21
Lymphoma	2.29 (24)	4.94	3.08 (33)	4.07
Myeloma	2.58 (27)	1.72	1.68 (18)	1.41
Oesophagus	3.24 (34)	3.27	1.31 (14)	1.63
Pancreas	4.01 (42)	2.70	4.48 (48)	2.75
Stomach	1.81 (19)	2.32	2.15 (23)	1.26
Testis	0.19 (2)	1.34	–	–
Uterus	–	–	3.64 (39)	5.27
Brain	1.43 (15)	1.59	1.12 (12)	1.23
Colorectal	12.69 (133)	12.46	12.98 (139)	10.43
Lung	17.56 (184)	13.34	14.75 (158)	11.86
Ovary			4.20 (45)	4.32
Oral	1.34 (14)	3.07	0.75 (8)	1.64
Melanoma	1.53 (16)	4.30	2.24 (24)	4.45
Prostate	14.98 (157)	26.35		
Other	25.0 (262)	9.64	23.72 (254)	8.58
Total	100 (1074)	100	100 (1071)	100

### Symptoms

Supplementary table 3 shows the most frequently occurring symptoms in the 28 days before the index date, and cancer incidence in patients with normal and raised inflammatory markers with these symptoms. None of the symptoms identified are high-risk symptoms warranting urgent cancer referrals under current NICE guidelines. The commonest symptoms were abdominal symptoms, joint symptoms, infective symptoms, and non-specific symptoms. Cancer incidence was significantly higher for those with a raised vs. normal inflammatory markers in all symptom subgroups except patients with throat symptoms. PPVs were >5% for those with raised inflammatory markers associated with cough, back pain, nausea and vomiting, and chest pain.

## Discussion

This is the first study looking at the overall incidence of cancer in primary care following inflammatory marker blood testing. Primary care patients with a raised inflammatory marker have an overall one-year cancer incidence of 3.53%, more than twice the risk in those with a normal test. Cancer incidence rises with rising levels of inflammatory markers and is higher still if a second test shows persistent raised inflammatory markers. However, inflammatory markers are not a useful rule-out test for cancer, as with a sensitivity of 46.1% for CRP, 43.6% for ESR and 49.7% for PV, roughly half of the tested patients with cancer had a normal inflammatory marker test in the 1 year before diagnosis. Patients with *normal* inflammatory markers have a cancer incidence of 1.50%, higher than the untested group with 0.97% cancer incidence; this is because the mere fact that a test has been performed predicts cancer, and this increased risk is only partially mitigated by a negative test result. Importantly, women under 60 and men under 50 with raised inflammatory markers have a risk of cancer below 3% so investigations for cancers would not usually be warranted.

### Strengths and weaknesses of the study

The main strengths of this study are the large sample size and the setting in primary care, which is where the initial suspicion of cancer usually arises. Studies using CPRD are reliant on the quality of data recording, but as blood test results are electronically transmitted to GP records this reduces the risk of missing data or bias. GPs are likely to record diagnoses of cancer carefully, and the use of Cancer Registry Data also improved outcome ascertainment in the linked practices.

The main weakness of the study is the lack of full data on the reason for inflammatory marker testing. As a result, the sample is heterogenous, and will include patients with clinically apparent infections or autoimmune conditions. Sensitivity analysis, excluding patients with pre-existing autoimmune diseases and infections confirms that this does not make a clinically significant difference to the cancer incidence figures reported here. The benefit of looking at all tests, rather than restricting analysis to tests done for specific indications, is that it reflects the way that inflammatory markers are used in real-life clinical practice. Although GPs may not be looking for cancer specifically when they request inflammatory markers, they should consider the possibility of cancer when interpreting test results.

The primary outcome, incidence of cancer at 1 year, is a proxy measure for prevalence of cancer at the time of testing. This period was chosen as a reasonable compromise between identifying all cancers related to the raised inflammatory marker, but ideally omitting cancers unrelated to the test. Most of the cancers in the test positive group were identified within 1 year, with no excess cancer risk in the second year after testing. We could only obtain cancer registry data for ~55% of our patients, as some CPRD regions are not yet linked to cancer registry. We chose not to limit our analysis to only practices with linked data, as this could introduce bias. Sensitivity analysis restricting to only patients with cancer registry linkage suggests that our overall PPV estimates may be small underestimates, due to small numbers of missing cancers in the unlinked practices.

We chose to use a standardised definition of raised inflammatory markers to aid interpretation and generalisability. This conservative definition slightly reduced the proportion of test results defined as abnormal, but sensitivity analyses showed minimal impact on overall results compared to laboratory specified upper limits of normal. It is likely that the conservative thresholds we used reflect real-life clinical practice, with clinicians less likely to action peri-normal test results.

Our untested comparison group did not have inflammatory marker testing in 2014 but could have had tests before or afterwards; the alternative of selecting a comparison group never to have had an inflammatory marker test was likely to introduce a bias from particularly good health.

### Comparison with previous literature

Several old studies have examined cancer risk in patients with significantly raised inflammatory markers. For example in a cohort of 1004 hospital outpatients with an ESR > 100 mm/h, 17% had malignancy,^29^ while in another hospital cohort 16% of those with ESR > 100 mm/h had malignancy.^30^ In our cohort 13.6% of those with ESR > 100 mm/h developed cancer in one year, the slightly lower figure probably representing the primary care setting. More recently, CRP and ESR have been evaluated as a tool for predicting cancer in patients with non-specific symptoms referred to Diagnostic Outpatient Clinics for rapid access to cancer diagnostics in Denmark.^31^ In this setting cancer prevalence was much higher at 19.8%; those with raised CRP had an odds ratio of 1.41 for cancer, after adjustment for age and sex, similar to our adjusted DOR for raised CRP of 1.79. Our findings demonstrate that this association exists, not only in the highly selected group of patients referred with suspected cancer, but also in unselected primary care patients, where the initial triaging and referral decisions must be made. Although we found no evidence that CRP, ESR or PV were superior to one another in relation to overall cancer detection, this may not be true for all types of cancer; for example recent studies have shown that ESR and PV are superior to CRP for myeloma diagnosis.^32^ As myeloma is known to have a strong association with raised inflammatory markers, it is perhaps surprising that myeloma comprised only small proportion of the overall cancers (<3%); when these were excluded from the analysis the incidence of cancer in the raised inflammatory marker cohort was 3.45%, compared to 3.53% overall.

### Implications for clinical practice and research

Current UK NICE guidelines recommend urgent cancer referral for any patient with a risk of cancer of 3% or higher,^8^ with studies of patient preferences suggesting an even lower threshold of 1%.^33^ With overall PPVs of 3.53%, inflammatory markers may aid earlier diagnosis of cancer. Inflammatory markers are not currently part of NICE guidelines for cancer diagnosis, with the exception of myeloma. Our findings suggest that, whilst not a useful rule-out test, inflammatory markers may have a role as a triage test in patients with ‘low-risk but not no-risk’ symptoms of cancer.

Interpretation of inflammatory marker test results must take into account the reasons for testing; if there is a clinically obvious explanation for raised inflammatory markers from history and examination, then further investigations for cancer would not usually be appropriate. Women under 60 and men under 50 with raised inflammatory markers have a risk of cancer below the 3% threshold, and in the absence of other risk factors, further investigations for cancers would not usually be warranted. For older patients with unexplained raised inflammatory markers, our study supports a strategy of repeat testing, with lower cancer incidence in those for whom the test returns to normal, and higher cancer incidence in those with rising inflammatory markers. Further investigations for cancer should be considered in patients with persistent unexplained raised inflammatory markers, particularly in older men, who are at highest risk. With significantly raised inflammatory markers, especially if accompanied by ‘low-risk but not no-risk’ cancer symptoms or signs, urgent investigation or referral may be appropriate, without repeat testing. The range of possible cancers is wide, so the choice of further investigations will vary depending on the clinical history and examination findings; recently introduced multidisciplinary diagnostic centres for patients with non-specific but concerning symptoms,^34^ may be appropriate if a clear source cannot be found. Excluding myeloma is not sufficient, as this only contributes a small proportion of the cancers diagnosed in the raised inflammatory marker cohort.

Although the PPVs from a raised inflammatory marker are clinically significant, inflammatory markers have poor sensitivity, so cannot be used to rule-out cancer. This is discordant with qualitative work suggesting that most doctors use inflammatory markers as a rule out test.^7^ The clinical usefulness of this study is therefore in guiding the clinical management of a patient with a raised inflammatory marker, particularly when this has been used as a non-specific test. Inflammatory markers should be used judiciously for possible cancer, taking into account the risks of false positives, which may generate anxiety, and false negatives, which may generate inappropriate reassurance. Rather than ruling cancer in or out definitively, inflammatory markers function as a Bayesian test, with a positive result increasing the risk of cancer, and negative results decreasing cancer risk. Further research is needed to explore how inflammatory marker blood test results, combined with other common blood tests, such as platelet, haemoglobin and calcium levels, alongside symptoms and signs, could generate prediction models with increased accuracy, to improve the early diagnosis of cancer in primary care.

## Conclusions

Raised inflammatory markers are associated with cancer and may predate the diagnosis by several months, especially in older patients, male patients, and those with very high or persistent abnormalities. Appropriate and timely investigation of patients with raised inflammatory markers may help improve the early detection of cancer. Inflammatory markers are not a useful rule-out test for cancer.

## Supplementary information

Supplementary figures and tables

## Data Availability

This study is based on CPRD data and is subject to a full licence agreement which does not permit data sharing outside of the research team.
