# Supplementary material for: Predictive value of inflammatory markers for cancer diagnosis in primary care: a prospective cohort study using electronic health records
Source: Br J Cancer. 2019 Apr 24;120(11):1045–51. doi: 10.1038/s41416-019-0458-x (PMC6738065; doi:10.1038/s41416-019-0458-x)
Supplement: Supplementary file 1 — Supplementary figures and tables [file 41416_2019_458_MOESM1_ESM.docx]

**Supplementary Table 1: ESR upper limit of normal** (derived using the CPRD mean upper limit of normal stratified by age and gender, rounded to the nearest integer, in mm/hr)

|  | **Men** | **Women** |
| --- | --- | --- |
| **<40** | 11 | 14 |
| **40-49** | 12 | 15 |
| **50-59** | 14 | 17 |
| **60-69** | 14 | 18 |
| **70-79** | 20 | 22 |
| **>80** | 20 | 23 |

**Supplementary Table 2: Sensitivity analyses**

Cancer incidence in those with raised inflammatory markers, normal inflammatory markers and untested controls, in selected subgroups.

| **Subgroup** | **Cancer incidence % (95% CI)** | | |
| --- | --- | --- | --- |
|  | **Raised inflammatory markers** | **Normal inflammatory markers** | **Untested** |
| Full cohort  (n=194,777) | 3.53 (3.37-3.70) | 1.50 (1.43-1.58) | 0.97 (0.87-1.07) |
| Restricting to patients with ≥1 year follow up in CPRD (n=158,025) | 3.87 (3.67-4.06) | 1.60 (1.52-1.68) | 1.02 (0.91-1.13) |
| Using laboratory specified upper limit of normal (n=194,777) | 3.38 (3.22-3.54) | 1.50 (1.43-1.58) | 0.97 (0.87-1.07) |
| Restricting to patients eligible for cancer registry linkage (n=110,245) | 3.82 (3.58-4.05) | 1.63 (1.53-1.73) | 1.04 (0.90-1.17) |
| Restricting to patients without pre-existing autoimmune disease or infection^[[1]](#footnote-1)^ in the past 1 month (n=174,500) | 3.70 (3.51-3.89) | 1.52 (1.44-1.60) | 0.94 (0.85-1.04) |
| Excluding patients with myeloma diagnosis (n=194,698) | 3.45 (3.28-3.62) | 1.48 (1.41-1.56) | 0.95 (0.86-1.05) |

**Supplementary Table 3:** Top 20 most frequently occurring symptoms in the 28 days before the index date, and cancer incidence in patients with normal and raised inflammatory markers with these symptoms*****

|  | **Normal inflammatory marker cancer incidence % (95% CI)** | **Raised inflammatory markers cancer incidence % (95% CI)** | **p-value** |
| --- | --- | --- | --- |
| Abdominal pain (n=10,011) | 1.49  (1.20-1.77) | 4.97  (4.17-5.77) | <0.001 |
| Tiredness (n=8,332) | 1.10  (0.85-1.36) | 2.84  (2.09-3.59) | <0.001 |
| Cough (n=5,801) | 2.03  (1.54-2.51) | 5.29  (4.33 – 6.25) | <0.001 |
| Joint pain (n=3,678) | 1.31  (0.87 – 1.75) | 2.34  (1.45 – 3.24) | 0.023 |
| Diarrhoea (n=3,463) | 1.01  (0.60 – 1.42) | 3.11  (2.09 – 4.12) | <0.001 |
| Headache (n=3,451) | 0.96  (0.58 – 1.35) | 2.07  (1.12 – 3.02) | 0.012 |
| Back pain (n=3,141) | 1.81  (1.24 – 2.37) | 6.21  (4.57 – 7.84) | <0.001 |
| Lower back pain (n=2,937) | 1.81  (1.23 – 2.38) | 5.32  (3.71 – 6.93) | <0.001 |
| Dizziness (n=2,854) | 1.58  (1.04 – 2.11) | 3.85  (2.37 – 5.34) | <0.001 |
| Pain generalised (n=2,832) | 1.19  (0.70 – 1.69) | 3.42  (2.23 – 4.60) | <0.001 |
| Chest pain (n=2,700) | 1.83  (1.22 – 2.44) | 6.13  (4.41 – 7.84) | <0.001 |
| Knee pain (n=2,597) | 0.56  (0.20-0.93) | 2.42  (1.37 – 3.47) | <0.001 |
| Rash (n=2,456) | 0.86  (0.41-1.32) | 2.84  (1.61-4.08) | <0.001 |
| Urinary tract infection (n=2,334) | 2.27  (1.44-3.11) | 4.56  (3.21-5.90) | 0.003 |
| Nausea & vomiting (n=1,812) | 1.66  (0.92-2.40) | 5.47  (3.67-7.26) | <0.001 |
| Shoulder pain (n=1,803) | 0.99  (0.41-1.57) | 4.70  (4.07-15.7) | <0.001 |
| Malaise (n=1,691) | 1.11  (0.45-1.76) | 3.43  (2.05-4.82) | 0.001 |
| Throat symptoms (n=1,660) | 0.79  (0.24-1.33) | 1.64  (0.57-2.71) | 0.12 |
| Chest infection (n=1,579) | 1.82  (0.84-2.81) | 4.61  (3.09-6.12) | 0.003 |
| Low mood (n=1,415) | 0.94  (0.32-1.55) | 3.13  (1.30-4.95) | 0.004 |

*Frequency of symptoms in untested patients was too low to allow calculations of cancer incidence in symptomatic subgroups

**Supplementary Figure 1: Polynomial logistic regression of cancer incidence against inflammatory marker test result as a continuous variable**

1. Jessica Watson. CRPD codes: infections and autoimmune conditions. University of Bristol Data Repository. DOI: https://doi.org/10.5523/bris.2954m5h0ync672u8yzx16xxj7l [↑](#footnote-ref-1)
